# Epigenetic Editing of *Ascl1* Gene in Neural Stem Cells by Optogenetics

**DOI:** 10.1038/srep42047

**Published:** 2017-02-09

**Authors:** Chiao-Ling Lo, Samrat Roy Choudhury, Joseph Irudayaraj, Feng C. Zhou

**Affiliations:** 1 Department of Anatomy & Cell Biology, Indiana University School of Medicine, Indianapolis, IN, USA; 2 Bindley Bioscience Center, Department of Agricultural & Biological Engineering, Purdue University, West Lafayette, IN, USA; 3 Stark Institute of Neuroscience Research, Indiana University School of Medicine, Indianapolis, IN, USA.

## Abstract

Enzymes involved in epigenetic processes such as methyltransferases or demethylases are becoming highly utilized for their persistent DNA or histone modifying efficacy. Herein, we have developed an optogenetic toolbox fused to the catalytic domain (CD) of DNA-methyltransferase3A (DNMT3A-CD) or Ten-Eleven Dioxygenase-1 (TET1-CD) for loci-specific alteration of the methylation state at the promoter of *Ascl1 (Mash1)*, a candidate proneuron gene. Optogenetical protein pairs, *CRY2* linked to DNMT3A-CD or TET1-CD and *CIB1* fused to a Transcription Activator-Like Element (TALE) locating an Ascl1 promoter region, were designed for site specific epigenetic editing. A differentially methylated region at the *Ascl1* promoter, isolated from murine dorsal root ganglion (hypermethylated) and striated cells (hypomethylated), was targeted with these optogenetic-epigenetic constructs. Optimized blue-light illumination triggered the co-localization of TALE constructs with DNMT3A-CD or TET1-CD fusion proteins at the targeted site of the *Ascl1* promoter. We found that this spatiotemporal association of the fusion proteins selectively alters the methylation state and also regulates gene activity. This proof of concept developed herein holds immense promise for the ability to regulate gene activity via epigenetic modulation with spatiotemporal precision.

DNA methylation defines the epigenetic state of the genome and has been associated with the transcriptional state of genes^1^. To date, a vast number of studies have aimed to measure DNA methylation levels as a means of gauging gene transcription and appraising biological status and disease conditions. However, beyond the relative terms of hypermethylation and hypomethylation, very less empirical information is available regarding the causal relationship between DNA methylation levels, transcriptional factor binding efficiency and eventual turnover of the transcriptional machinery. For example, questions relating to the effect of site-specific DNA methylation on the transcriptional state of a gene or the efficacy of transcriptional regulation on the distance between DNA methylation clusters (e.g. at CpG island distance) and the transcription start site (TSS) is unknown. More importantly, clarifying the role of DNA methyltransferases (DNMT) or Ten-Eleven Translocation dioxygenase (TET) enzymes in editing the DNA methylation marks in a site-specific manner or spanning CpG sites is critical for epigenetic programming. Our study aims to investigate this query by testing the feasibility of epigenetic editing and characterizing the key parameters of DNA methylation in relationship to transcription using an optogenetically-equipped epigenetic editing tool that functions at a gene promoter at a loci-specific resolution.

We adopted a novel approach that uses epigenetic editing with optogenetic tools to control gene expression. This approach involves the recruitment of epigenetic modifiers, comprising of DNMT3A or TET1, guided by Transcription Activation like Elements (TALE) to the selective methylation or demethylation sites at the promoter of suppressed or inactive regulatory genes^2^,^3^. In particular, we report on our optogenetically engineered platform for site-specific epigenetic interventions of the *Ascl1* gene (see below) by using the blue-light inducible dimerizing protein pair cryptochrome-2 and its interacting protein CRY2-CIB1 to ensure site and enzyme specificity and to monitor their role on gene expression.

*Ascl1 (Mash1*) is a proneuron gene essential for neuronal lineage and formation in the developing brain following the neuroprogenitor state^4^. Multipotent neural stem cell (NSC) differentiation into neurons and glial cells are subject to epigenetic regulation and transcription^5^. To test the aforementioned paradigm, we used two sets of rat NSCs, the rat dorsal root ganglion (DRG) and striatal (STR) NSCs^6^,^7^, with contrasting DNA methylation profiles at the promoter of the *Ascl1* gene. In DRG NSCs, the *Ascl1* gene is minimally methylated (also see Results) throughout the gene and has previously been shown to preferentially yield neurons^6^,^7^, while in STR NSCs, *Ascl1* is methylated and preferentially yields glial cells^8^.

In our previous study, we demonstrated that selective epigenetic editing at the subtelomeric regions using optogenetic toolbox may significantly contribute to the telomeric length homeostais^9^. The optogenetic regulatory platform developed herein is demonstrated to selectively alter the endogenous epigenetic status of a proneuron gene namely *Ascl1* in a site-specific manner, enabling the process of regulating transcription of *Ascl1*. We analyzed both DNMT3A and TET1, which modified DNA methylation in opposite directions at a distance of ~40 bp distance from their targeted binding sites. Targeted epigenetic alterations, even to a lesser extent, at 4 CpG sites in a strategic ~30 bp multi-transcription factor binding site was sufficient to regulate the transcription of *Ascl1*. Based on our findings, we found that both the level of DNA methylation and location of DNA methylation modifiers from the target sites are critical to transcriptional regulation, which is discussed as a highlight of the report.

## Results

### *Ascl1* methylation and gene expression profile

**Figure 1:**
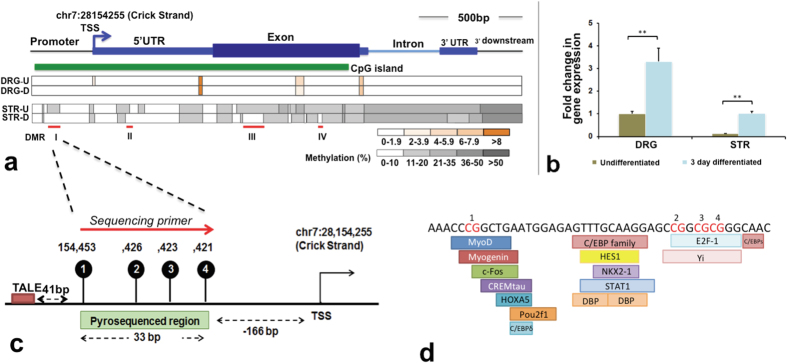
DNA methylation and gene expression of rat *Ascl1* in DRG and STR NSCs. **(a)** The gene structure of *Ascl1* is shown on top of the figure. The dark blue box represents the exon, light blue boxes are untranslated regions (UTR), the blue line represents the intron, arrow indicates the direction of transcription, and the green box is CpG island. DNA methylation profiles for DRG and STR NSCs at both undifferentiated and 3-day differentiated stages (n = 3) were laid out corresponding to the gene structure. We sequenced a 2.3 kb region that spans the *Ascl1* gene body and promoter regions (chr7: 28152247-28154486). The color of the box indicates the level of DNA methylation per the scale bar at the bottom of the figure. Red lines indicate statistically significant DMRs (p < 0.001) between STR-U (undifferentiated STR) and STR-D (3-day differentiated STR). (**b**) Relative gene expression level of *Ascl1* in undifferentiated and 3-day differentiated DRG and STR neural stem cells (n = 4). The relative expression of *Ascl1* was calculated using the ΔΔCt value. The average Ct values of target genes were normalized to the average Ct value of the internal control gene *18 s* to calculate ΔCt, and the ΔΔCt value was generated by normalizing the ΔCt value of each cell line to the ΔCt value of the undifferentiated DRG. The p-values between any two groups, except for undifferentiated DRG and differentiated STR, were all below 0.001. **(c)** Pyrosequenced region in the DMR-I of *Ascl1* promoter contained 4 CpG sites. The first CpG site is 41 bp upstream of the TALE binding region. The sequenced region is 166 bp from the transcription start site (TSS) of *Ascl1* **(d)** Predicted transcription factor binding sites in the sequenced region. **P < 0.01.

Bisulfite amplicon sequencing at single base pair resolution of the entire *Ascl1* gene including the 300 bp of the promoter region was conducted for the native DNA methylation profiles of the DRG NSCs and STR NSCs (Supplementary Table 1). We found that the DNA methylation profile for *Ascl1* differs significantly between the two types of NSCs. The methylation levels of *Ascl1* in DRG NSCs were low, mostly <10%, throughout the gene body and at its promoter, in both undifferentiated and differentiated states. In contrast, the CpG methylation levels of *Ascl1* in STR NSCs were much higher, in the range of between 30–50%; the highest level of methylation was observed at the 3′ end of the gene body and gradually decreased from the 3′ end to the 5′ end in the promoter in the undifferentiated state. General STR NSC methylation was decreased at the CpG island (p < 0.01) in differentiated state as compared to the undifferentiated state (Fig. 1a). We found that, at the extent of CpG island, there are four CpG clusters of differentially [DNA] methylated regions (DMRs) in the *Ascl1* gene between undifferentiated and differentiated NSCs. The DMR-I is in the promoter, DMR-II is in the 5′UTR, and DMR-III and DMR-IV are in the exon (Fig. 1a); each DMR has 3–4 CpGs. For reasons indicated in next section, DMR-I was chosen as target for the methylation modification (see next section DMR Targeting). Furthermore, the distal DMR-III or IV was examined to demonstrate the range and specificity of TET and DNMT enzymatic effect. The minimally methylated DRG NSCs were targeted with DNMT3A, while the highly methylated STR NSCs were targeted with TET1.

We further found that the differential DNA methylation level between the two types of NSCs were also present with differential levels of *Ascl1* expression, as obtained from a qRT-PCR analysis (Fig. 1b). In the undifferentiated state, the *Ascl1* promoter was hypomethylated in DRG NSCs and hypermethylated in STR NSCs. Concurrent with our prediction, DRG NSCs expressed an 8.3-fold greater in the expression level of *Ascl1* compared to STR NSCs. Furthermore, STR NSCs exhibited decreased DNA methylation in the DMR regions (Fig. 1a), and increased *Ascl1* expression on the third day of differentiation (Fig. 1b). These data cumulatively suggest a possible correlation between differential levels of DNA methylation to distinct gene expression between DRG and STR NSCs.

### DMR and DMR targeting

The DMR-I was chosen for testing our hypothesis involving site-specific epigenetic modifications (Fig. 1a) for the following reasons: (a) it contains the most significant methylation difference between DRG and STR NSCs, (b) it is in the promoter region, and (c) there are many (about 20) transcription factor (TF) binding sites at this DMR. DMR-I consists of four CpG sites (Rn5 Chr7: 28154421-28154454) in the *Ascl1* promoter, and is 166 bp upstream of the TSS (Fig. 1c), which also contains several putative transcription factor-binding sites, including c-Fos, HES1 and E2F-1 (Fig. 1d) [obtained from the PROMO database (http://alggen.lsi.upc.es/)^10^,^11^]. To target the DMR-I, we designed the TALE-locator to bind to a specific site, 41 bp upstream (chr7: 28154495-28154514) of the DMR-I region. The reasons for this consideration are as follows—(a) to avoid the possibility of targeted epigenetic enzymes masking the TATA box critical to the RNA polymerase binding, and (b) to determine the enzymatic coverage of nucleotide length by the TALE DNA binding module (Fig. 1c).

### Construction and expression of optogenetic fusion protein

**Figure 2:**
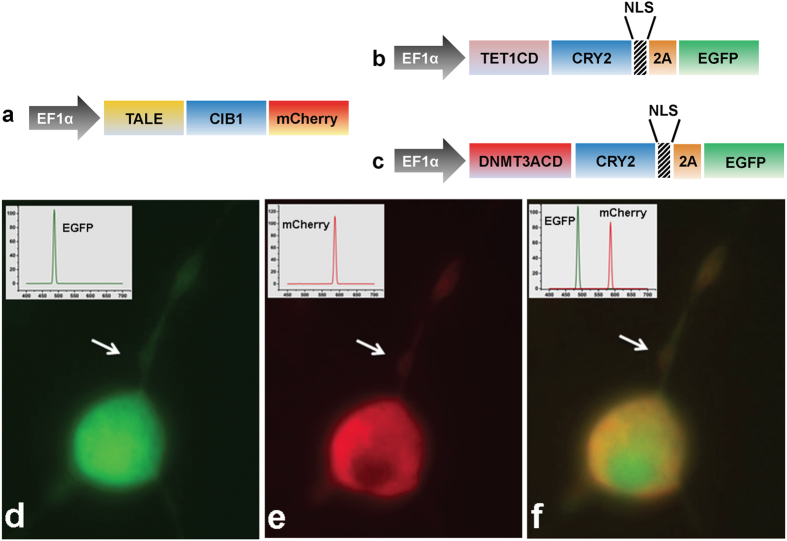
*Ascl1* promoter targeting constructs. **(a)** Scheme of the TALE-CIB1 fusion protein (~172 kD) containing mCherry as the fluorescent marker. (**b**) Scheme of the TET1-CD-CRY2-EGFP (TET1_-CRY2-EGFP)_ fusion protein (175 kD). (**c**) Scheme of the DNMT3A-CD-CRY2-EGFP (DNMT3A_-CRY2-EGFP_) fusion protein (128 kD). (**d–f**) Representative co-expressing TET1_-CRY2-EGFP_ fusion protein (**d**, green) and TALE-CIB1-mcherry fusion protein (**e**, red) with merged image (**f**, orange and green) in a 2 day differentiated rat neuronal stem cell. We observed that TALE-CIB1 fusion proteins were predominantly expressed in the cytoplasm, while TET1_-CRY2-EGFP_ fusion proteins were expressed throughout the whole cell body and axon. White arrow: axon. EF1a: Elongation factor 1-alpha promoter; NLS: nuclear localization signal; EGFP: Enhanced Green Fluorescent Protein.

Two set of constructs, the site-specific DNA binding module (TALE module targeting upstream of DMR-I) and the epigenetic effector modules (DNMT3A- or TET1), were made for the desired editing of DNA methylation (see Designing the optogenetic constructs in the Methods Section). The native TALE module was designed to target a 20 bp site, upstream to the DMR-I, and was fused to the N-terminus of optically inducible cryptochrome-2-interacting binding protein-1 (CIB1) protein to form the TALE-CIB1 fusion protein (~172kD), which also contained mCherry as a fluorescent reporter (Fig. 2a). In contrast, the epigenetic effector constructs were generated by fusing the catalytic domain of TET1 or DNMT3A to the N-terminus of CIB1 interacting protein cryptochrome-2 (CRY2) with the fluorescent reporter Enhanced Green Fluorescent Protein (EGFP), resulting in a **TET1**_**-CRY2-EGFP**_construct (~175 kD) for selective demethylation and a **DMNT3A**_**-CRY2-EGFP**_construct (~128 kD) for methylation (Fig. 2b,c). TALE-CIB1 fusion proteins were co-transfected with either TET1_-CRY2-EGFP_ or DNMT3A_-CRY2-EGFP_ constructs, with similar transfection efficiency in both NSCs (~40% in the DRG and ~35% in STR NSCs) (Fig. 2f). After co-transfection with the locator (TALE-CIB1) and effectors (TET1_-CRY2-EGFP_ or DNMT3A_-CRY2-EGFP_), we observed the excitation spectra of EGFP, or mCherry expression from the individually transfected cells or a combination of both the fluorophores from the co-transfected cells (Fig. 2d–f). We also observed the characteristic fluorescence emission spectra of EGFP and mCherry in the cell-free extract of the transfected cells (**Inset; 2d-2f**). From the fluorescent micrographs, we observed that TALE-CIB1 fusion proteins were predominantly expressed in the cytoplasm (Fig. 2e), while the epigenetic fusion proteins (TET1_-CRY2-EGFP_ or DNMT3A_-CRY2-EGFP_) were expressed throughout the cells (Fig. 2d). The localization of fusion proteins was in agreement with a previous report^12^.

### Optogenetic induction of Site-specific methylation

**Figure 3:**
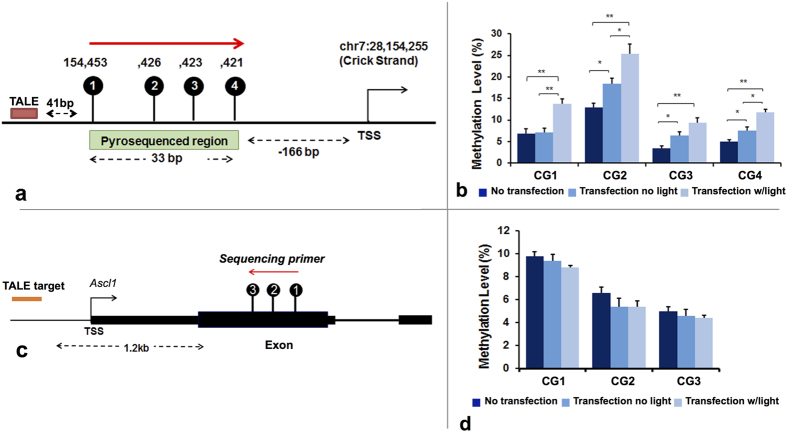
Optogenetic induction of site-specific methylation in DRG cells using DNMT3A_-CRY2-EGFP_. **(a)** Pyrosequenced region at the TALE target sites of the *Ascl1* promoter. The first CpG site is 41 bp upstream of the TALE binding region. The sequenced region is 166 bp from the transcription start site (TSS) of *Ascl1* **(b)** DNA methylation level of DNMT3A_-CRY2-EGFP_ and TALE co-transfected DRG cells in the sequenced region illustrated in (**a**). **(c)** Pyrosequenced region at the non-TALE targeting site. This sequenced region is 1.2 kb upstream of TALE target sites, and contains 3 CpG sites. Methylation in non-TALE target region was accessed in **(d)** DNMT3A_-CRY2-EGFP_ and TALE co-transfected DRG cells. The extent of changes in methylation was compared between Non-transfected, transfected with no light, and transfection with light groups. Student’s paired t-test with two tails was used for statistical analysis. Data are presented as the mean ± SEM. N = 5 for each group. *p < 0.05; **p < 0.01.

**Figure 4:**
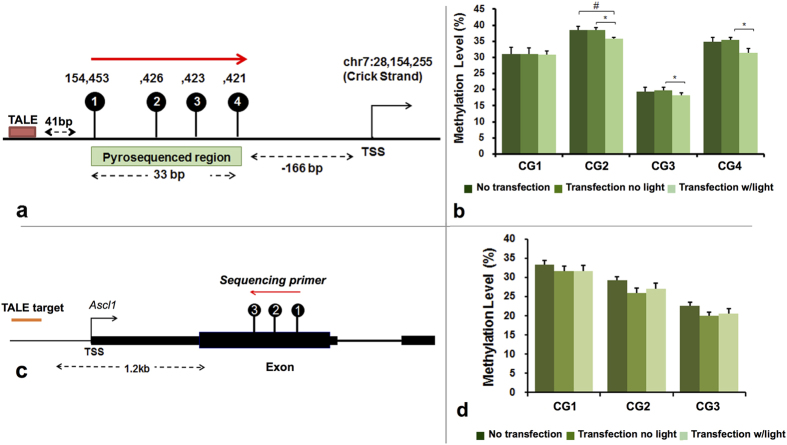
Optogenetic induction of site-specific methylation in STR cells using TET1_-CRY2-EGFP_. **(a**) Pyrosequenced region in the TALE target sites **(b)** DNA methylation level of TET1_-CRY2-EGFP_ and TALE co-transfected STR cells in the sequenced region illustrated in (**a**). (**c**) Pyrosequenced region in non-TALE targeting site. Methylation in non-TALE target region was accessed in (**d**) TET1_-CRY2-EGFP_ and TALE co-transfected STR cells. The extent of changes in methylation was compared between No-transfection, transfection no light, and transfection with light. Student’s paired t-test with two tails was used for statistical analysis. Data presented as mean ± SEM. N = 5 for each group. ^#^P < 0.1; *P < 0.05; **p < 0.01.

The changes in the levels of targeted methylation at the DMR-I of the *Ascl1* promoter were further confirmed via pyrosequencing of the same region in both DRG (Fig. 3b) and STR (Fig. 4b) NSCs. The DRG harbored low levels of methylation (between 3–13%, Fig. 3b) and was used for methylation editing using DMNT3A_-CRY2-EGFP_ (Fig. 3b), while STR exhibited characteristically high levels of methylation (between 20–38%, Fig. 4b) and was used for demethylation editing using TET1_-CRY2-EGFP_ (Fig. 4b).

Following blue light illumination (15 mW/cm^2^) for 3 hr, the co-transfected DRG NSCs with DMNT3A_-CRY2-EGFP_ and TALE fusion proteins exhibited a significant increase in methylation level at three of the four CpG sites (CpG1, CpG2, and CpG4) in DMR-I as compared to the transfected but non-light treated groups (Transfection no light) (Fig. 3b). Quite effectively, all four CpGs in the co-transfected with light illumination (transfection w/light) DRG NSCs exhibited significant increase in methylation compared to the no-transfection group. In contrast, methylation in the designed off-target CpG sites, which were 58 bp upstream of DMR-IV, were not affected by the above transfections or light-stimulation (Fig. 3c,d).

In the demethylation editing study, the STR NSCs that were co-transfected with TET1_-CRY2-EGFP_ and TALE fusion proteins (Fig. 4) and underwent illumination for 1 hour exhibited a significant decrease in methylation level at CpG2, CpG3, and CpG4 compared to the co-transfected, non-illuminated cells (Transfection no light) (Fig. 4b). Methylation in the co-transfected, non-illuminated cells, however, did not significantly differ from the no transfection groups. Similar to the study in DRG, the decrease in methylation in STR was only observed in the targeted DMR and absent from the off-target CpG sites near DMR-IV (Fig. 4c,d). We found that DNA methylation showed the biggest changes, optimized at 12-hour post-illumination as compared to 4-hour and 18- hour post-illumination recovery (Supplementary Figure 1). Therefore, the downstream analysis was assessed 12 hour after illumination. In STR cells, the cytotoxicity increased when illumination was over 2 hours, and the methylation change was more significant in cells with 1-hour of illumination compared to cells with 2-hour illumination; therefore, we used 1-hour light exposure as the optimized time of illumination.

### Site-specific induction was associated with altered *Ascl1* gene expression

**Figure 5:**
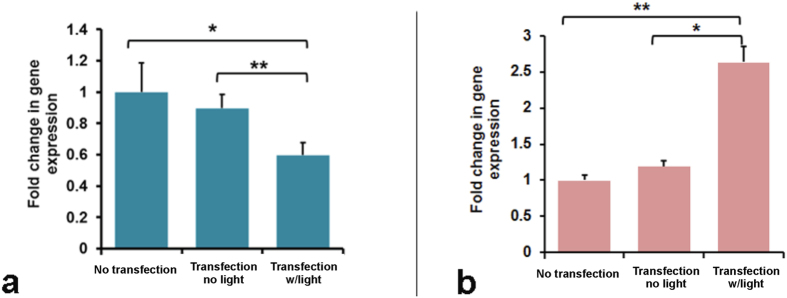
Relative gene expression of *Ascl1* in **(a)** DRG NSC co-transfected with DNMT3A_-CRY2-EGFP_ and TALE **(b)** STR NSC co-transfected with TET1_-CRY2-EGFP_ and TALE. The relative expression was calculated based on the ΔΔCt value. The average Ct values of target genes were normalized to the average Ct value of the internal control 18 s to calculate ΔCt, and the ΔΔCt value was generated by normalizing the ΔCt value of Transfection without light or Transfection with light group to the ΔCt value of the No-transfection group. Student’s paired t-test with two tails was used for statistical analysis. Data presented as mean ± SEM. *p < 0.05; ** p < 0.01. n = 5 for each group.

Next, we determined the gene expression level of *Ascl1,* followed by epigenetic editing. Our results showed that, for the DRG NSCs, the transfection w/light group exhibited a site-specific increase in DNA methylation, and also resulted in a decrease in *Ascl1* expression (Fig. 5a). Additionally, the decrease in DNA methylation previously observed in the STR transfection w/light groups resulted in a 2.5-fold increase in *Ascl1* expression (Fig. 5b). In contrast, no significant difference in gene expression was observed between the No-transfection and Transfection no light groups in both DRG and STR NSCs, indicating that a change in *Ascl1* expression was possibly due to the synergistic action of locator and effector constructs followed by blue-light illumination.

### Altered NeuN protein expression was associated with Optogenetic induction

**Figure 6:**
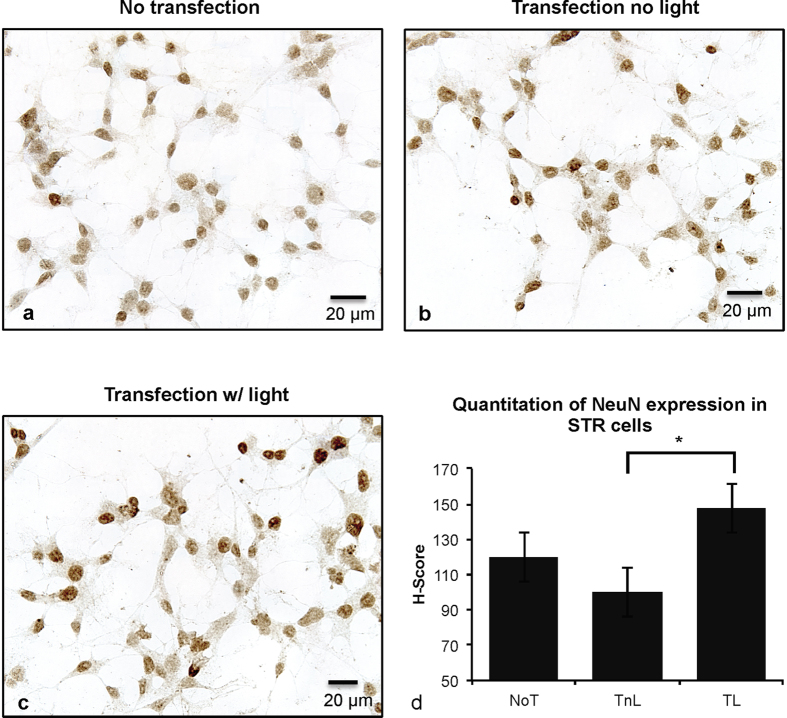
Protein expression of NeuN in **(a)** STR NSC no transfection control **(b)** STR NSC co-transfected with TET1_-CRY2-EGFP_ and TALE without light treatment (**c**) STR NSC co-transfected with TET1_-CRY2-EGFP_ and TALE with light treatment (**d**) Quantitate of the immunostaining images by H-score, where higher H-score represents higher protein expression among all the cells analyazed in the frame. Total eight images from four biological samples were analyzed for each group. All images were taken under the same parameters and magnitude (40X). Student’s t-test was used for statistical analysis. Data are presented as mean ± SEM. *P < 0.05; NoT: no transfection; TnL: Transfection no light; TL: Transfection with light.

We further examined the protein expression of the neuronal protein marker NeuN by immunocytochemistry (ICC) to determine whether the differentiation potential of NSCs could also be altered upon optogenetic modulation at the *Ascl1* promoter. STR transfection w/light cells, which exhibited increased *Ascl1* expression, were expected to have a higher potency to differentiate into neurons and a lower potency to differentiate into glial cells, and we expected the opposite effects in DRG transfection w/light group. We quantified the ICC signal intensities from individual cells using the H-score method^13^. Based on our cumulative data, we observed a significant (p = 0.02) induction of NeuN expression in the STR transfection w/light cells comparing to the STR transfection without light group (Fig. 6). This indicates that site-specific editing of *Ascl1* is leading alteration in neural stem cell fate determination.

## Discussion

### Design of Epigenetic Editing Tool

**Figure 7:**
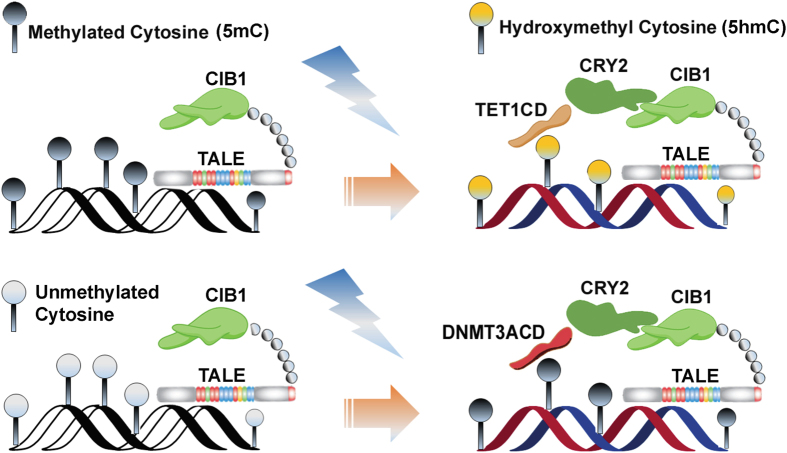
Schematic showing that the pair of fusion protein constructs—containing either the catalytic domain of TET1 (top panel) or DNMT3A (bottom panel), while the target construct contained the TALE-CIB1 fusion protein. Each of the constructs contained an optogenetic component, CRY2 or CIB1, endowed with a blue-light inducible/reversible association involving effector-locator coupled to enable epigenetic editing. The association of TALE-CIB1 with TET1-CD-CRY2 induces conversion of 5mC into 5hmC or subsequent demethylation, while the association of TALE-CIB1 with DNMT3A-CD induces the addition of methyl group to unmethylated cytosines.

Advancing beyond the Zinc-finger protein-targeted DNA editing since 2000^14^, transcription-like effector nucleases (TALENs)^15^ and the Clustered regularly interspaced short palindromic repeats (CRISPR)/Cas (a nuclease) system^16^,^17^,^18^ are being used for targeted cleavage of DNA for genetic editing. The concept of *epigenetic editing* is more complex than *genetic editing* not only because editing is upstream to the transcription, but also because epigenetic editing adds a few tiers of uncertainty, including the complexity of epigenetic enzymes, off-targeted binding due to their affinity to CpGs, functional epigenetic sites (location and range) and functional threshold of epigenetic changes. Nevertheless, targeted enzymatic modification such as DNA methylation is popular not only because of its potential to alter the epigenetic state but also offers a means to better understand the causal mechanism of epigenetic regulation on the transcription machinery. In epigenetic editing, the effectors (e.g. DNMTs, TETs, histone methyltransferases…etc.) are preferred to be fused to a DNA binding modules such as TALE-TFs or dCAS9 rather than DNA cleavage enzymes (e.g. FokI or Cas9 endonucleases). For example, to use the CRISPR system for epigenome editing, Cas9 is deactivated (dCas9) and fused with epigenetic modifiers, such as DNMT3A or TET1^19^,^20^, and p300^21^, while guided RNA (gRNA) is used as the DNA binding module to manipulate the epigenome. For the present study, we decided to couple the optogenetic dimerizing protein pair CRY2 and CIB1 to the TALE over CRISPR-dCas9 mediated DNA binding modules (see Fig. 7). Previous reports have already reported a number of drawbacks of CRISPR-dCAS9 system, despite of their facile construction strategy and cost effectiveness. For example, the attachment of TAL-TF proteins to its target DNA binding site is more specific and dynamic, compared to dCAS9-TFs. This reduces the chance of non-specific (off-target) effects^22^. In contrast to dCAS9, TALE proteins could be directly fused to the effector domains, which allows for multiple targeting of distinct effectors including epigenetic modifiers across multiple loci^23^. In addition, a recent study demonstrated that TALE proteins causes higher enrichment of activating components (p300) and active histone markers (H3K27Ac) at the enhancer of *Oct4* and *Nanog* gene loci, compared to CRISPR-dCAS9 system, in the course of gene reactivation^24^. We provided a comparison of the tailor-use of TALE with the use of CRISPR-dCAS9 in Table 1. This Table will provide options for the investigators when choosing an optimal method for their own use.

Furthermore, the use of single fusion protein of TALE-TET for DNA demethylation^2^, TALE-DNMT for methylation^25^, or TALE- histone acetyltransferase/deacetylase for histone mark modification^12^ have been reported for epigenetic editing. With single fusion proteins, the effect of the epigenetic enzyme is uncontrolled and constitutive (i.e. continuous). In comparison, with optogenetic constructs, we can periodically bring the epigenetic effector fusion protein to the target site. This provides us with a controlled and regulatory approach of epigenetic regulation. In this study, we demonstrated a site-specific modulation of DNA methylation at the *Ascl1* promoter by introducing a pair of fusion protein constructs—containing either the catalytic domain of DNMT3A or TET1, while the target construct contained the TALE-CIB1 fusion protein that was inducible for 5mC/5hmC conversion or demethylation (Fig. 7). Each of the constructs contained an optogenetic component, CRY2 or CIB1, endowed with a blue-light inducible/reversible association involving effector-locator coupling, and enables site-specific epigenetic editing at spatiotemporal precision. Additionally, since success of co-transfection is critical for site-specific epigenetic editing, each of the constructs also contained a fluorescent protein marker (mCherry or EGFP) to report the presence and efficiency of the co-transfection.

### Targeted Epigenetic Editing

In our study, we conducted site-specific epigenetic editing by TALE-targeting at a specific genomic location fused to methylation modifiers that were precisely coupled with optogenetic control. The fluorescent protein markers, incorporated in these constructs facilitated visualization of their intracellular co-transfection. Interestingly, DNMT3A_-CRY2-EGFP_ or TET1_-CRY2-EGFP_ were initially diffused throughout cytoplasm, but begin to accumulate in the nucleus upon blue light exposure, which probably also facilitate intranuclear coupling to their light mediated partner.

A concern relating for targeted editing using constitutive promoter-driven expression of effectors (DNMTs or TETs) that arose was that the binding nature of the effector would cause the transfected targeting construct to act non-specifically throughout many other off-target regions, rendering DNA methylation non-specific if the effector and locator is designed to yield one fusion protein. To nullify this possibility and ensure precise targeting, we adopted two approaches, such as (a) constructing two fusion proteins with optogenetic coupling or (b) developing only the catalytic (without the binding) domain of DNMT3A and TET1, which reduces the chance of their binding to the native off-target sites. The precise genomic targeting utilizing TALE is able to achieve DNA methylation modification specifically at 4 CpG sites at the proximal DMR-I (41 bp downstream) to the TALE-binding site, but not at CpG sites near DMR-IV (~1.2 kb downstream) to the TALE-binding site. Both hypermethylation by transfected DMT3A_-CRY2-EGFP_ and demethylation by transfected TET1_-CRY2-EGFP_ demonstrated the proximal (Figs 3b and 4b) over the distal effect (Figs 3d and 4d) of epigenetic modifiers, and together validated the specificity of targeted epigenetic editing. In addition, we believe that through this system of paired optogenetic editing, a high resolution of epigenetic action at functional epigenetic sites (e.g. CpG sites) can be identified, responsible for the consequential transcription.

### Functional Methylation Site

We assess the minimal CpG methylation alterations and the potential location of these changes that might affect the transcription. With our optogenetic approach, we demonstrated that alteration of as few as 4 CpGs spanning ~30 bp DMR-I region was sufficient to alter the transcription of *Ascl1*. This was achieved by validating the proximal and distal CpG alterations and monitoring the transcriptional changes. Methylation with site-directed DNMT3A_-CRY2-EGFP_ not only decreased *Ascl1* gene expression, but also reduced demethylation. In contrast, site-directed TET1_-CRY2-EGFP_ increased *Ascl1* gene expression, and decreased methylation in this DMR-I. Finally, phenotypic analysis of NeuN protein expression (a neuronal marker) also confirmed the possibility of site-specific epigenetic editing guiding neural specification. To our knowledge, this is the first demonstration of a combination of an optogenetic system with a site-specific DNA methylation editing platform to provide temporal control of the manipulation of DNA methylation using the TALE system in neuronal genes.

At least 15 transcriptional factor (TF) binding sites, including those of Hes1, Nkx2-1, HOXA5, E2F-1, MyoD, C/EBP family, STAT1, and c-Fos (see Fig. 1d), were predicted spanning the 30 bp sites including 4 CpGs. Given the binding partners of TFs and their individual functions, the picture of epigenetic regulation of transcription is likely more complex. For example, the DMRs, which involve enhancers at far upstream of promoters, may influence promoter regulation. DMRs in the gene body, such as in intronic regions or intron-exon junctions, may also affect alternative splicing if the splicing binding protein is epigenetically regulated. These “Functional Methylation Site” (FMS) may exist in each gene and may serve as a switch for the transcription. We advocate that FMS as small as of 3–4 CpGs in DMR critical to TFs binding could respond to environmental input and subject to transcriptional changes.

### DNA methylation Threshold

By using a passive DNA demethylase (TET1) and DNA methyltransferase (DNMT3A), we showed that DNA methylation could either be increased or decreased at the same sites. With methylation altered by DNMT3A_-CRY2-EGFP,_ we found an average of a 1.58-fold (1.38 to 1.92) increase in DNA methylation across all four CpG sites upon treatment with light, which was associated with a 1.75-fold reduction in *Ascl1* gene expression. In contrast, when TET1_-CRY2-EGFP_ was used in the STR cells, an average of 1.1-fold decrease in DNA methylation was observed. Interestingly, although the change in DNA methylation by TET1_-CRY2-EGFP_ is smaller than that by DNMT3A_-CRY2-EGFP,_ we found a greater change in gene expression, which is ~2.6-fold increase, by TET1_-CRY2-EGFP._

One thing to note is that bisulfite pyrosequencing cannot differentiate 5-methylcytosine (5mC) from 5-hydroxylcytosine (5hmC). Given that TET1 converts 5mC into 5hmC, and that 5hmC can lead to gene activation, we noticed the TET-1 induced demethylation is rather modest as compared with the prominent expression changes of *Ascl1*. It is likely that a combination of demethylation and a transformation for 5hmC occurred at this DMR. Also, it is possible that some of the methylation levels we detected in the STR co-transfected with light group were contributed by 5hmC, which likely is a result of the action of TET1_-CRY2-EGFP._ The 5hmC component and its contribution will be studied in a large yield of stem cells in our laboratory.

Of all the editing studies, we have seen a slight increase of methylation in part of the CpGs only by the DNMT construct with transfection but not light stimulation (Fig. 3b). Its elevation is quite small as compared to those with light stimulation. Nevertheless, we documented its occurrence. This is likely an efficient transfection resulted in undirected binding to the target site. Furthermore, the same TALE construct coupled with TET1CD did not resulted in methylation changes unless light stimulation was performed.

### Catalytic Range of DNA (De)methylation Enzymes

Our designed TALE bound to a region of *Ascl1* promoter (that is 240 bp to 260 bp upstream of the TSS), altered methylation of 4 CpG sites, which were 41 bp to 74 bp downstream of TALE binding sites (or −166 to −199 bp from TSS). This range of enzymatic activity is supported by previous finding demonstrating that the distance of altered methylation from target DNA sites was somewhere between 20 bp to 200 bp^2^,^25^. In our study, we only measured DNA methylation on the Watson strand. It is expected that similar levels on both strands as Maeder *et al*. demonstrated in their study^2^. Another interesting point, is that the enzymatic activities of TET1_-CRY2-EGFP_ and DNMT3A_-CRY2-EGFP_ are likely to function at both ends of TALE target site due to a linker inserted in the fusion protein constructs that enables TET1_-CRY2-EGFP_ or DNMT3A_-CRY2-EGFP_ to flip between both ends. However, we were unable to measure methylation upstream of TALE binding sites because that region is relatively CG dense (5 CpGs in 20 bp), resulting in difficulties in primer design and sequencing.

We found reduced cell viability in STR cells after longer blue light exposure. The cytotoxicity was observed mainly in TET1_-CRY2-EGFP_ transfected cells but not in DNMT3A_-CRY2-EGFP_ transfected cells. We have tried up to 3-hour light exposure for DNMT3A and have not observed similar finding. The cause is not clear, but apparently not due to the blue light. This effect may be specific to the catalytic domain of TET1. We have previously tried to transfect the full size construct of TET1 and DNMT3A, however the transfection efficiency of those were extremely low, therefore we pursue with TET1CD. Due the low transfection efficiency, at this point we cannot determine the toxicity effect between catalytic domain vs full length TET1. We may generate mutated catalytic domain as a comparison in the future.

In conclusion, epigenetic editing is far more complex than genetic editing, with multi-level considerations including the sites of action. Understanding how minimal changes in DNA methylation at identified strategic sites lead to gene expression not only facilitates a better understanding of how environmental input may “conveniently” alter an epigenetic “red button” to regulate the transcription of a specific gene, but also opens the door to learning more about how gene transcription can be harnessed through epigenetics. Our study, which used an optogenetic platform to demonstrate that a small DMR with a few CpGs in the promoter region of the *Ascl1* gene can be edited to change gene expression, helps to improve the understanding of targeted DNA methylation editing as an important component of epigenetic editing.

## Methods

### MiSeq profiling of *Ascl1* DNA methylation

Three samples each, for both undifferentiated and 3-day differentiated and DRG and STR cells, were used for methylation profiling of the entire *Ascl1* gene, including 300 bp of the promoter region (RGSC 5.0/rn5 chr7: 28152246- 28154536). Extracted genomic DNA was treated with sodium bisulfite using the EZ DNA Methylation-Direct™ Kit (Zymo Research, Irvine, CA, Cat # D5020). We designed primers to amplify sequences ≤ 600 bp using the Zymo Bisulfite Primer Seeker program (http://www.zymoresearch.com/tools/bisulfite-primer-seeker) and minimized the number of amplicons (Primers as listed in Supplementary Table 2). PCR amplification was carried out using hot-start DNA polymerase (AmpliTaq Gold, Life Technologies, Cat # 4311816). After amplification, all products were purified using DNA Clean & Concentrator™-5 (Zymo Research, Irvine, CA, Cat #D4004) or other similar purification methods and quantified using a Turner Biosystems TBS-380 Mini-Fluorometer (Turner Biosystems, Sunnyvale, CA) with a fluorescent nucleic acid stain (Quant-iT PicoGreen dsDNA Assay Kit, Life Technologies, Cat # P7589) for quantitating double-stranded DNA to ensure that equimolar amounts were added for the Nextera XT sample preparation (Illumina, San Diego, CA) step. Each sample was given a unique index during enzymatic fragmentation and library preparation, according to standard protocols (Illumina, San Diego, CA). Different amplicons from each sample were pooled prior to Taqmentation, as each amplicon is distinguishable by sequence analysis. The pooled library was loaded into the MiSeq platform (Illumina, San Diego, CA) according to the Nextera XT protocol. MiSeq™ Reagent Kit v2 was used for the run.

### Data analysis

The filtered Fastq data generated from MiSeq was analyzed at the Purdue Genomics Core Facility to trim off the index and adapter sequence. The CLC Genomics Workbench (Qiagen, Redwood City, CA) was used for further analysis. We used the completely methylated and fully unmethylated sequences as references. We then mapped reads of each sample to both references under the following parameters: similarity fraction = 0.9, length fraction = 0.5, nonspecific map-ignored. The rest of the parameters were set to default values. The mapped files were used for quality-based variant detection at frequency = 1%. For a CpG dinucleotide, if the Cytosine (C) was methylated, C was detected during variant detection. If the C was not methylated, it was converted into Uracil (U) and then Thymine (T) during PCR; therefore, the nucleotide was referred to as T. We then determined the % methylation at each site for each sample by calculating the frequency of C to T.

### Designing the optogenetic constructs

We modified the vector backbone of two light inducible (optogenetic) complementary fusion protein constructs, obtained from Addgene plasmid repository (https://www.addgene.org/) and originally contributed by F. Zhang group. A full length (native) Transciption Activator Like Element (TALE) was designed to target the chosen site at the *Ascl1* promoter (Fig. 7). The TALE module was synthesized by Genecopoeia, USA. The synthesized TALE along with a HA tag was then fused to the N-terminus of CIB1 (cryptochrome-interacting basic-helix-loop-helix) protein in the vector (#47458, Addgene). In addition, a mCherry coding sequence was fused to the C-terminus of the CIB1 sequence in the same vector. The optogenetic complement of the TALE fusion protein contained either the catalytic domain of TET1 (TET1-CD) or DNMT3A (DNMT3A-CD), fused to the N-terminus position of the cryptochrome-2 (CRY2) photolyase homology region (CRY2PHR)-EGFP fusion protein in the vector template (#47457). Inserts were incorporated into the vector backbone with a standard restriction-digestion based method. 100 ng of DNA were briefly PCR-amplified (CloneAmp HiFi PCR Premix (Clontech Laboratories Inc.) from their source plasmids, according to manufacturer’s instructions. PCR-amplified inserts and the vector template were then digested with restriction endonucleases followed by gel purification using the QIAEX II gel extraction kit (QIAGEN). The purified vector and inserts were ligated with the requisite amount of T4 DNA ligase buffer and enzyme system (New England Biolabs) and kept at room temperature for 15 minutes. The ligated product was then transformed into stellar competent cells (Clonetech Laboratories Inc.) and plated out on an Ampicillin (Amp) supplemented LB agar plate. Suitable clones were propagated in LB-Amp+ media and the plasmids were extracted with QIAprep Spin Miniprep Kit (QIAGEN). The full-length nucleotide sequence of the fusion proteins can be found in the supplementary information (supplementary sequence 1–3). The fusion protein was sequenced against a panel of primers as summarized in Supplementary Table 3, as well as sequencing primers (Supplementary Table 4) can be found in the supplementary results.

### Cell culture, transfection, and illumination

Rat neural stem cells (NSCs) were isolated from the dorsal root ganglion (DRG) and striatum. This study utilized rat dorsal root ganglion (DRG)-derived NSCs and striatum (STR)-derived NSCs that were previously established and characterized in our laboratory as a bona fide NSC^7^,^8^. Multipotency and stability were tested and the epigenetic profiles were characterized in these screened NSCs^7^,^26^. DRG NSCs were previously shown to preferentially yield neurons^6^,^7^ while STR NSCs preferentially yielded non-neuronal glial cells^8^. Adult NSCs were maintained in Dulbecco’s Modified Eagle Medium/F-12 Nutrient Mix (DMEM/ F-12) media containing N2 supplement (12 μL/mL, Life Technologies, Grand Island, NY), and penicillin-streptomycin (12 μL/mL, Sigma, St. Louis, MO) and grown in a humidified incubator at 37 °C and 5% CO2. Media was supplemented with 10 ng/mL epidermal growth factor (EGF, Harlan Bioproducts for Science, Indianapolis, IN) and basic fibroblast growth factor twice a week (bFGF, PeproTech, Rocky Hill, NJ) for maintenance of NSCs in the neurosphere form. During medium changes throughout the years, no passaging (trypsin digestion and cell transferring, except dividing into multiple flask) was performed. All neurospheres for analyses were screened by size <1 mm shape (round neurosphere) for their robust growth.

For transfection, the undifferentiated neurospheres were mechanistically dissociated into small spheres via pipetting. Approximately 10^6^ cells were used for one transfection. 100 ul of rat neural stem cell nucleofector kit (Lonza, Walkersville, MD), plus 5 ug of each plasmid constructs were used for co-transfection. The transfection was performed using a Nucleofector 2b device (Lonza, Walkersville, MD) under program A-31 follow manufacture’s instruction. After 24 hours, the transfected cells were placed in fresh, warm culture medium, and assessed for expression of the construct under a fluorescent microscope. About 48 hours after transfection, the cells were seeded in a laminin (50 μg/mL, Sigma) coated 35 mm culture dish, and supplemented with differentiation medium consisting of Neurobasal media supplemented with 10% fetal bovine serum, 1.2% B27 and 1.2% penicillin-streptomycin. 24 hours after differentiation, the cells were treated with a mounted high-power LED blue light (455 nm) (Thorlabs), controlled by a DC2100 driver (Thorlabs), under the power of 15 mA (~0.6 mW/cm^2^) for 1 hour for TET1/TALE co-transfected STR cells, and 3 hours for DNMT3A/TALE co-transfected cells. 12-hour post-light treatment, the cells were collected by scraping from the Petri dish and were snap-frozen for DNA/RNA extraction.

### RNA extraction and gene expression

Total RNA was isolated from each cell line using Qiagen RNeasy Mini Kit (Qiagen, Valencia, CA). An on-column DNA digestion was performed during RNA purification using the Qiagen RNase-Free DNase Set (Qiagen, Valencia, CA). RNA quality and quantity was assessed using a Nanodrop spectrophotometer. 1 ug of RNA from each cell line was converted into cDNA using an ABI High-Capacity cDNA Reverse Transcription Kit (Life Technologies, Grand Island, NY). 100 ng of cDNA was used as a template for qRT-PCR in combination with a TaqMan® Gene Expression Master Mix (Life Technologies, Grand Island, NY) and Taqman Gene-specific probes (Assay ID: *Ascl1*—Rn00574345_m1; 18 S—Rn03928990_g1; S100b—Rn04219408; Rbfox3—Rn01464214_m1) on the StepOnePlus™ Real-Time PCR System (Life Technologies, Grand Island, NY). We assayed a minimum of four biological replicates for each group; cycling reactions were performed in duplicate. The relative expression of each gene was calculated based on the ΔΔCt value, where the results were normalized to the average Ct value of data from the *18* *S* housekeeping gene. We used Student’s paired t-test with two tails for statistical analysis. Data presented as mean ± SEM.

### DNA extraction and Pyrosequencing

Genomic DNA was extracted from frozen cells with the DNeasy blood and tissue kit (Qiagen, Fremont, CA). 600 ng of the extracted genomic DNA was bisulfite converted, using the EZ DNA Methylation-Gold™ Kit (Zymo Research, Irvine, CA). Bisulfite-biotinylated primer specific PCR was carried out using the PyroMark PCR kit (Qiagen, Fremont, CA) follows manufacturer’s instruction. PCR products were pyrosequenced using PyroMark Q24 system (Qiagen) and Pyromark Q24 advanced CpG reagents (Qiagen, Fremont, CA) against region-specific primers (Supplementary Tables 5 and 6).

### TF binding prediction

To predict putative transcription factor binding of the TALE target site in *Ascl1* promoter region, sequences of the TALE target region was retrieved from the RGSC Rnor_5.0 rat genome assembly and were used as input to the PROMO database (http://alggen.lsi.upc.es/)^10^,^11^, which uses TRANSFAC for prediction. We presented the output from PROMO using the search criteria for species of Muridae (includes both mouse and rat).

### Immunocytochemistry

The transfected and untransfected cells were subplated into 16-well chamber slides (ThermoFisher, Waltham, MA) coated with poly-D-lysine (20 mg/mL, Sigma, St. Louis, MO) and laminin (50 μg/mL, Sigma, St. Louis, MO), and followed by the same culture condition and light treatment as described previously. 12 hours post-light treatment, cells were fixed in 4% paraformaldehyde fixative for 24 hours, and then replaced with PBS.

Immunostaining procedures followed a routine procedure as previously established for stem cell cultures in our laboratory^8^,^26^. In brief, endogenous peroxide was quenched with 3% H_2_O_2_ and 1% Triton X-100 was applied for 30 minutes to permeabilize the cell membranes, followed by incubation with 2 N HCl. Non-specific binding was blocked using 1.5% goat serum, plus 0.1% Triton X-100 in PBS. Primary antibody, rabbit α-NeuN, 1:250 (Cell Signaling Technology, Denvers, MA was incubated overnight at room temperature in the blocking buffer. The chamber wells were then incubated for 90 min in goat anti-rabbit IgG secondary antibodies conjugated with biotin (Jackson ImmunoResearch, West Grove, PA) followed by rabbit Peroxidase-conjugated-streptavidin (Jackson ImmunoResearch, West Grove, PA) tertiary antibody for 90 min. The immunostaining was visualized by incubation of 0.05% 3′-3′ diaminobenzidine (Sigma-Aldrich, St. Louis, MO) prepared at 1 μg/1 mL and 1 mg/1 mL 3% H_2_O_2_ for 10 min, followed by a methyl green counterstaining. The slides were then imaged using a Leica Digital Microscope 6000B, Leica DC500 camera, and Firecam Imaging Software (version 1.7.1) (Leica Microsystems, Buffalo Grove, IL).

In order to quantify the staining intensities, ImageJ 1.48 (NIH, Bethesda, MD) was used. Representative wells were selected for each treatments and the intensity of each cell in the frame was measured at 40X objective. The image was first processed using the Color Deconvolution plugin (Gabriel Landini, 2007) with a similar protocol to Ruifrok *et al*.^27^. Then, cellular regions were selected using the ellipse tool. Cells that were not fully in the frame, had overlapping regions with other cells, or were in a cluster were omitted. The mean intensity of each region was used for comparison, and the H-score for each frame was calculated using the formula^13^


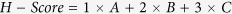


where A is the percentage of cells weakly stained, B is the percentage of cells moderately stained, and C is the percentage of cells strongly stained. The H-score formula thus gives the immunostaining a score from 0 to 300 while giving the cells with higher staining intensity higher weight.

## Additional Information

**How to cite this article**: Lo, C.-L. *et al*. Epigenetic Editing of *Ascl1* Gene in Neural Stem Cells by Optogenetics. *Sci. Rep.* **7**, 42047; doi: 10.1038/srep42047 (2017).

**Publisher's note:** Springer Nature remains neutral with regard to jurisdictional claims in published maps and institutional affiliations.

## Supplementary Material

Supplementary Information

## Figures and Tables

**Table 1:** Comparison between TALE-TF and CRISPR0-dCas9 in the use for epigenetic editing.

	TALE-TF	CRISPR-dCas9	Ref.
Type of recognition	Protein-DNA	RNA-DNA	
Specificity	More specific and little off-target activity	Higher tolerance to mismatches, less specific	22
Effector conjugation	TALE could be directly fused with effector domain	Effector could not be conjugated with sgRNA	23
Enrichment of effector at target site	Feasible and efficient	Not designed for	24
Complication with unwinding effect of DNA	Not yet reported	Yes, eg. Enhancer	24
Control of expression	For both methods, the expression is more constitutive. Upon co-transfection of dCAS9 fused epigenetic proteins such as TET1 or DNMT3A and the single-guide RNAs (sgRNAs), there is always a possibility of uncontrolled and continuous epigenetic effect. The same is also true for the TAL fused epigenetic proteins.
