# Supplementary material for: Epigenetic Editing of Ascl1 Gene in Neural Stem Cells by Optogenetics
Source: Sci Rep. 2017 Feb 9;7:42047. doi: 10.1038/srep42047 (PMC5299429; doi:10.1038/srep42047)
Supplement: Supplementary Information [file srep42047-s1.pdf]

Supplementary information

Epigenetic Editing of *Ascl1* Gene in Neural Stem Cells by Optogenetics

Chiao-Ling Lo[1], Samrat Roy Choudhury[3], Joseph Irudayaraj[3], Feng C. Zhou[1,2]

[1]Department of Anatomy & Cell Biology, Indiana University School of Medicine

[2]Stark Institute of Neuroscience Research, Indiana University School of Medicine

[3]Agricultural & Biological Engineering, Purdue University

Supplementary Figure 1

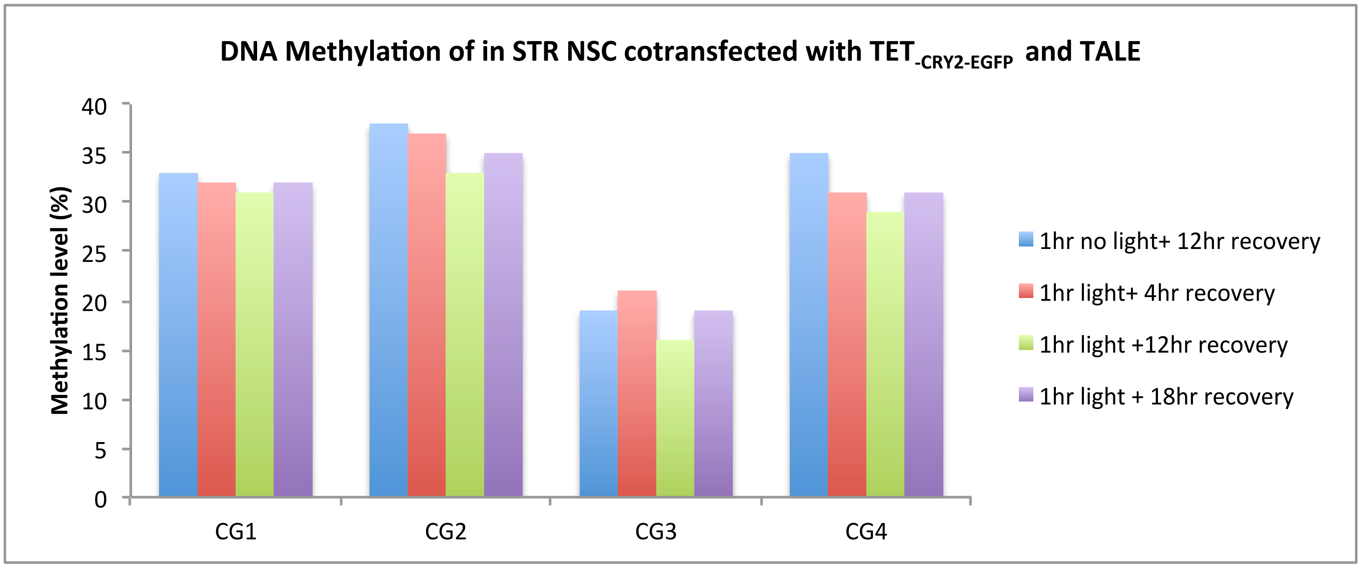

Supplementary Figure 1. Comparison of DNA methylation levels in STR neural stem cells at different timeframes post-light treatment. $TET1_{-CRY2-EGFP}$ and TALE co-transfected STR cells were divided into 4 groups for the following treatment: (1) no light treatment with 12 hour recovery, (2) 1-hour light treatment followed by 4 hour, (3) 12 hour, (4) 18 hour recovery before cell isolation to access the duration of DNA methylation changes. Methylation levels were assessed by pyrosequencing in the TALE target sites.

Ascl1 CpG methylation

Location	3'end								End of exon2	
CpG No.	1	2	3	4	5	6	7	8	9	10	11 new CpG
Genomic position (Rn 5.0) Chr7	28152246	28152248	28152270	28152303	28152334	28152351	28152362	28152394	28152411	28152424	28152471
DRG undiff-1	<1	<1	1.43	<1	<1	<1	<1	<1	<1	<1	<1
DRG undiff-2	<1	<1	<1	<1	<1	<1	<1	<1	<1	<1	<1
DRG undiff-3	<1	<1	<1	<1	<1	<1	<1	<1	<1	<1	<1
DRG diff-1	<1	<1	<1	<1	1.13	<1	<1	<1	<1	<1	<1
DRG diff-2	<1	<1	<1	<1	<1	<1	<1	<1	2.09	1.92	<1
DRG diff-3	<1	<1	<1	<1	<1	<1	<1	<1	<1	<1	<1
striatal undiff-1	43.33	46.71	40.67	38.89	37.64	32.98	34.14	33.10	37.80	31.68	19.60
striatal undiff-2	41.42	46.67	39.42	36.75	39.91	33.27	33.61	33.27	36.57	33.44	21.58
striatal undiff-3	44.30	46.93	42.29	39.88	42.27	37.73	37.00	38.80	39.52	36.12	22.95
STR und Ave	43.02	46.77	40.79	38.51	39.94	34.66	34.92	35.06	37.97	33.74	21.38
striatal diff-1	31.53	35.07	35.19	29.04	24.40	24.37	25.66	22.75	38.06	39.45	11.30
striatal diff-2	36.13	37.95	34.11	30.85	33.78	36.95	29.58	28.78	45.05	36.36	29.43
striatal diff-3	46.15	46.15	48.08	43.15	46.97	38.78	41.70	35.21	47.18	38.67	26.83
STR Diff Ave	37.94	39.72	39.13	34.35	35.05	33.37	32.31	28.91	43.43	38.16	22.52
T-test STR und:dif	0.313	0.101	0.733	0.410	0.505	0.800	0.626	0.204	0.131	0.050	0.852

Methylation level	p-value
0-10%	p<0.05
10-20%	0.05<p<0.07
20-35%	
35-50%	
>50%	

		intron 1				exon1					
12	13	14	15	16	17	18	19	20	21	22	23
28152560	28152563	28152637	28152843	28152845	28152881	28153037	28153049	28153052	28153065	28153071	28153080
1.06	<1	<1	<1	<1	<1	8.46	<1	<1	<1	<1	<1
<1	<1	<1	<1	<1	<1	8.35	<1	<1	<1	1.63	<1
<1	<1	<1	<1	<1	<1	5.34	<1	<1	<1	1.27	<1
<1	<1	<1	<1	<1	<1	6.02	<1	<1	<1	<1	<1
<1	<1	<1	1.17	<1	5.91	5.89	<1	1.99	<1	<1	<1
<1	<1	<1	<1	<1	<1	7.26	<1	1.41	<1	<1	<1
15.23	27.49	32.29	22.81	21.16	23.38	19.73	21.70	24.94	22.43	37.09	25.06
17.52	24.41	35.38	26.18	25.00	27.29	11.07	9.47	12.42	12.20	21.97	12.10
20.38	31.43	36.31	27.74	26.13	27.19	21.76	21.15	22.95	23.59	32.65	21.96
17.71	27.77	34.66	25.58	24.10	25.95	17.52	17.44	20.10	19.41	30.57	19.71
9.99	14.79	20.13	23.74	19.45	28.39	20.19	15.59	18.97	14.53	22.83	13.34
9.77	14.88	35.18	23.90	22.16	27.01	17.85	11.33	15.36	16.80	20.48	11.10
17.44	27.28	44.51	26.24	25.63	26.80	16.01	12.47	17.14	15.86	22.55	11.29
12.40	18.98	33.27	24.63	22.41	27.40	18.02	13.13	17.16	15.73	21.95	11.91
0.144	0.130	0.857	0.599	0.511	0.354	0.893	0.361	0.505	0.374	0.131	0.121

24	25	26	27	28	29	30	31	32	33	34	35
28153097	28153125	28153133	28153137	28153151	28153158	28153163	28153169	28153188	28153190	28153192	28153202
<1	<1	<1	<1	<1	<1	<1	<1	<1	<1	<1	<1
<1	<1	<1	<1	<1	<1	<1	<1	<1	<1	<1	<1
<1	<1	<1	<1	<1	<1	<1	<1	<1	<1	<1	<1
<1	<1	<1	<1	<1	<1	<1	<1	<1	<1	<1	<1
<1	<1	<1	<1	<1	<1	<1	<1	<1	<1	<1	<1
<1	<1	<1	<1	<1	<1	<1	<1	<1	<1	<1	<1
25.43	21.27	22.22	23.34	24.59	22.79	18.91	24.00	13.59	12.52	14.17	21.63
13.88	12.84	13.57	13.77	16.64	15.15	9.32	12.77	7.73	6.68	7.90	13.49
21.34	20.64	22.39	24.29	23.61	23.32	19.85	20.88	12.31	12.31	12.31	21.28
20.22	18.25	19.39	20.47	21.61	20.42	16.03	19.22	11.21	10.50	11.46	18.80
14.13	12.06	12.37	19.58	23.72	23.87	18.31	19.64	9.40	9.40	9.40	14.27
17.69	13.00	14.61	14.92	21.27	17.60	13.45	17.53	8.11	8.11	8.11	12.07
14.20	15.25	17.65	17.87	17.79	16.90	10.95	14.15	9.20	7.46	9.93	14.17
15.34	13.44	14.88	17.46	20.92	19.46	14.24	17.11	8.90	8.33	9.15	13.50
0.244	0.169	0.242	0.453	0.832	0.794	0.678	0.600	0.274	0.337	0.298	0.127

36	37	38	39	40	41	42	43	44	45	46	47
28153206	28153209	28153213	28153218	28153250	28153253	28153270	28153306	28153312	28153316	28153318	28153322
<1	<1	<1	<1	<1	<1	5.91	<1	1.09	<1	<1	<1
<1	<1	<1	<1	<1	<1	3.96	<1	<1	<1	<1	<1
<1	<1	<1	<1	<1	<1	3.94	<1	<1	<1	<1	<1
<1	<1	<1	<1	<1	<1	1.66	<1	<1	<1	<1	<1
<1	<1	<1	<1	<1	<1	3.13	<1	<1	<1	<1	<1
<1	<1	1.07	<1	<1	<1	5.58	<1	<1	<1	<1	<1
21.80	23.82	20.32	22.73	12.90	15.46	29.42	19.06	20.13	16.64	15.30	19.25
16.15	15.92	12.53	16.44	4.32	9.03	18.75	11.90	15.99	10.46	8.36	9.99
22.23	24.35	19.73	22.48	12.90	15.24	28.82	18.25	19.67	18.66	17.15	19.68
20.06	21.36	17.52	20.55	10.04	13.24	25.66	16.41	18.60	15.25	13.60	16.31
15.03	14.18	10.11	10.42	7.02	9.90	21.27	12.42	13.15	9.02	7.78	13.03
11.88	10.89	12.60	10.37	8.04	7.95	20.02	15.20	15.81	11.61	10.76	11.86
13.57	15.22	12.77	14.38	8.25	9.21	21.11	12.81	16.27	12.20	8.52	13.21
13.49	13.43	11.83	11.72	7.77	9.02	20.80	13.48	15.08	10.95	9.02	12.70
0.038	0.058	0.098	0.023	0.475	0.125	0.235	0.293	0.097	0.180	0.180	0.321

48	49	50	51	52	53	54	55	56	57	58	59
28153327	28153330	28153332	28153337	28153344	28153370	28153390	28153393	28153402	28153412	28153422	28153426
<1	<1	<1	<1	<1	<1	<1	<1	<1	<1	<1	<1
<1	<1	<1	<1	<1	<1	<1	<1	<1	<1	<1	<1
<1	<1	<1	<1	<1	<1	1.17	<1	<1	<1	<1	<1
<1	<1	<1	<1	1.08	<1	<1	<1	<1	<1	<1	<1
1.37	<1	<1	<1	1.15	<1	<1	1.04	<1	<1	<1	<1
<1	<1	<1	<1	<1	<1	<1	<1	<1	<1	<1	<1
15.97	4.72	5.45	32.29	35.00	25.90	19.48	14.40	18.64	16.95	14.59	13.46
10.99	3.37	3.22	19.41	21.45	15.46	10.77	7.34	9.98	6.82	8.16	7.08
14.13	4.31	7.20	30.76	33.57	24.13	17.00	13.56	16.31	13.77	12.82	10.94
13.70	4.13	5.29	27.49	30.00	21.83	15.75	11.77	14.98	12.51	11.85	10.49
6.29	4.48	4.77	18.35	19.05	17.43	9.77	5.94	8.62	12.83	9.98	10.11
7.00	1.58	4.16	17.98	20.64	20.08	15.23	11.51	15.36	15.86	16.12	14.74
10.35	2.09	4.12	18.03	22.32	16.39	11.53	6.74	11.97	9.37	6.69	5.42
7.88	2.72	4.35	18.12	20.67	17.97	12.17	8.06	11.98	12.69	10.93	10.09
0.039	0.223	0.467	0.082	0.101	0.320	0.306	0.260	0.408	0.964	0.797	0.908

60	61	62	63	64	65	66	67	68	69	70	71
28153432	28153449	28153463	28153481	28153485	28153491	28153503	28153506	28153518	28153521	28153545	28153547
<1	<1	<1	<1	<1	<1	<1	<1	<1	<1	<1	<1
<1	<1	<1	<1	<1	<1	<1	<1	<1	<1	<1	<1
<1	<1	<1	<1	<1	<1	<1	<1	<1	<1	<1	<1
<1	2.43	<1	<1	<1	<1	<1	<1	<1	<1	<1	<1
<1	<1	<1	<1	<1	<1	<1	<1	<1	<1	<1	<1
<1	1.10	<1	<1	<1	<1	<1	<1	<1	<1	<1	<1
16.00	16.78	16.57	17.20	14.72	20.50	19.80	14.66	18.15	12.92	17.72	15.75
8.69	6.84	8.03	9.02	6.82	9.76	11.04	7.62	9.90	7.56	7.61	8.15
13.25	14.83	16.99	16.11	11.88	17.37	16.91	13.04	16.38	12.11	15.17	12.02
12.65	12.82	13.86	14.11	11.14	15.88	15.92	11.78	14.81	10.86	13.50	11.97
11.21	6.84	8.41	6.64	6.20	3.53	2.48	2.22	7.80	2.00	1.67	1.68
17.76	18.59	12.24	13.58	6.59	14.54	16.24	5.19	20.51	15.09	19.99	18.59
5.56	7.34	7.14	7.84	4.70	7.38	4.51	4.00	3.41	4.13	8.76	5.37
11.51	10.92	9.26	9.35	5.83	8.48	7.74	3.81	10.57	7.08	10.14	8.55
0.796	0.719	0.235	0.228	0.089	0.178	0.178	0.026	0.499	0.436	0.613	0.573

72	73	new CpG	74	75	76	77	78	79	80	81	82
28153560	28153566	28153569	28153572	28153575	28153578	28153581	28153583	28153587	28153590	28153592	28153596
<1	<1	<1	<1	<1	<1	<1	1.19	<1	<1	<1	<1
<1	<1	<1	<1	<1	<1	<1	<1	<1	<1	<1	<1
<1	<1	<1	<1	<1	<1	<1	<1	<1	<1	<1	<1
<1	1.74	<1	<1	<1	<1	<1	<1	<1	<1	<1	<1
<1	<1	<1	<1	<1	<1	<1	<1	<1	<1	<1	<1
<1	1.49	<1	<1	<1	<1	<1	<1	<1	<1	<1	1.28
15.97	15.07	21.2734864	15.54	17.21	10.68	13.35	12.12	10.42	9.39	6.08	20.89
10.24	7.00	13.450012	9.19	9.81	5.81	6.64	6.64	7.34	4.47	2.94	9.59
13.84	15.11	18.059519	16.13	16.25	10.18	11.03	9.81	11.23	9.21	5.15	19.34
13.35	12.39	17.59	13.62	14.42	8.89	10.34	9.52	9.66	7.69	4.72	16.61
	6.95	7.12334524	6.87	1.67		1.37	6.77	6.04	10.01	1.00	5.49
23.39	22.36	21.89515	20.57	15.93	4.15	12.15	9.54	5.97	19.58	1.00	18.64
5.32	4.96	10.467033	6.83	7.02	6.00	3.89	3.89	3.97	7.39	1.00	7.44
14.35	11.42	13.16	11.42	8.20	5.08	5.80	6.74	5.32	12.33	1.00	10.52
0.896	0.881	0.427	0.688	0.262	0.169	0.299	0.288	0.034	0.315	0.016	0.324

5'UTR

83	84	85	86	87	88	89	90	91	92	93	94
28153617	28153632	28153641	28153652	28153658	28153701	28153703	28153713	28153733	28153753	28153761	28153768
<1	<1	<1	<1	<1	<1	1.28	<1	7.29	<1	<1	<1
<1	<1	<1	<1	<1	<1	<1	<1	16.71	<1	<1	<1
<1	<1	<1	<1	<1	<1	<1	<1	7.77	<1	<1	<1
<1	<1	<1	<1	<1	<1	1.37	<1	28.51	<1	<1	<1
<1	<1	<1	<1	<1	<1	<1	<1	6.21	<1	<1	<1
<1	<1	<1	<1	<1	<1	<1	<1	7.04	<1	<1	<1
12.30	17.93	15.31	11.88	18.22	6.87	18.25	9.74	16.10	8.87	15.82	10.48
5.58	8.74	6.72	6.37	9.20	3.60	4.86	4.34	11.96	6.59	15.02	9.50
10.36	14.96	14.11	11.94	17.68	7.43	9.58	10.08	17.02	6.25	14.06	8.59
9.41	13.88	12.05	10.06	15.04	5.96	10.90	8.05	15.03	7.24	14.97	9.52
2.00	7.70	8.25	2.28	6.98	3.13	5.34	5.13	10.77	3.53	11.69	5.90
7.38	12.86	9.14	10.16	16.05	5.17	5.17	6.13	13.03	8.95	16.03	15.25
4.74	6.04	6.65	4.87	4.99	2.83	5.50	3.25	9.69	4.81	13.05	5.66
4.70	8.87	8.02	5.77	9.34	3.71	5.33	4.83	11.16	5.76	13.59	8.94
0.136	0.214	0.221	0.221	0.273	0.183	0.229	0.190	0.104	0.465	0.374	0.864

95	96	97	98	99	100	101	102	103	104	105	106
28153781	28153812	28153837	28153839	28153870	28153892	28153905	28153908	28153930	28153933	28153935	28153945
<1	<1	<1	<1	<1	<1	<1	<1	<1	<1	<1	<1
<1	<1	<1	<1	<1	<1	<1	<1	<1	<1	<1	<1
<1	<1	<1	<1	<1	<1	<1	<1	<1	<1	<1	<1
<1	<1	<1	<1	<1	<1	<1	<1	<1	<1	<1	<1
<1	<1	<1	<1	<1	<1	<1	<1	<1	<1	<1	<1
<1	<1	<1	<1	<1	<1	<1	<1	<1	<1	<1	<1
13.00	13.06	6.97	9.83	14.43	8.41	9.16	1.00	5.37	11.67	7.87	13.18
12.45	10.64	4.67	8.96	9.31	6.76	8.71	7.56	4.26	6.95	6.27	7.53
11.41	10.42	5.04	7.67	11.26	7.29	8.37	7.69	3.32	5.56	6.80	9.79
12.28	11.37	5.56	8.82	11.67	7.49	8.74	5.42	4.32	8.06	6.98	10.16
6.99	4.09	6.40	6.40	7.01	5.87	6.95	5.47	1.42	3.00	1.00	2.16
13.52	12.20	3.16	3.16	9.12	1.00	1.00	1.00	1.85	9.26	1.00	5.30
7.19	8.40	3.24	3.24	9.52	3.31	4.96	4.26	4.12	4.81	2.89	5.15
9.24	8.23	4.27	4.27	8.55	3.39	4.30	3.58	2.46	5.69	1.63	4.20
0.237	0.276	0.372	0.021	0.138	0.051	0.065	0.515	0.145	0.418	0.002	0.037

107	108	109	110	111	112	113	114	115	116	117	118
28153954	28153956	28153961	28153965	28153967	28153973	28153977	28153980	28153983	28153986	28153994	28154029
<1	<1	<1	<1	<1	<1	<1	<1	<1	<1	<1	<1
<1	<1	<1	<1	<1	<1	<1	<1	<1	<1	<1	<1
<1	<1	<1	<1	<1	<1	<1	<1	<1	<1	<1	<1
<1	<1	<1	<1	<1	<1	<1	1.18	<1	<1	<1	<1
<1	<1	<1	<1	<1	<1	<1	<1	<1	<1	<1	<1
<1	<1	<1	1.11	1.31	<1	1.25	<1	<1	<1	<1	<1
6.91	6.18	5.84	9.05	5.33	13.23	13.11	10.77	16.13	11.95	12.35	9.42
6.52	3.60	6.68	6.42	4.90	9.96	11.00	10.36	11.08	8.27	9.64	8.81
5.11	3.54	5.27	7.58	4.91	9.99	10.42	10.04	15.83	9.66	12.88	7.70
6.18	4.44	5.93	7.68	5.05	11.06	11.51	10.39	14.35	9.96	11.62	8.64
1.15	1.15	1.00	4.41	1.56	5.82	4.82	9.14	11.22	5.06	6.45	4.83
1.94	1.94	2.68	7.33	7.33	8.09	4.69	4.41	4.65	5.60	8.20	4.35
1.74	1.74	1.50	2.81	2.81	5.57	5.89	6.31	5.52	4.54	6.25	5.70
1.61	1.61	1.73	4.85	3.90	6.49	5.13	6.62	7.13	5.07	6.96	4.96
0.002	0.035	0.003	0.137	0.549	0.028	0.002	0.053	0.052	0.012	0.017	0.005

119	120	121	122	123	124	125	126	127	128	129	130
28154041	28154044	28154048	28154063	28154068	28154074	28154081	28154091	28154095	28154176	28154188	28154201
<1	<1	<1	<1	<1	<1	<1	<1	<1	<1	<1	<1
<1	<1	<1	<1	<1	<1	<1	<1	<1	<1	<1	<1
<1	<1	<1	<1	<1	<1	<1	<1	<1	<1	<1	<1
<1	<1	<1	<1	<1	<1	<1	<1	<1	<1	<1	<1
<1	<1	<1	<1	<1	<1	<1	<1	<1	<1	<1	<1
<1	1.59	<1	<1	<1	<1	<1	<1	<1	<1	<1	<1
8.72	11.13	8.04	10.79	10.52	15.15	19.18	15.33	13.85	5.93	5.16	7.42
5.57	7.52	6.15	10.79	10.62	11.52	15.51	12.53	10.69	5.05	5.97	9.07
7.23	8.54	7.95	13.42	12.36	16.40	23.44	16.08	13.44	7.14	8.93	9.98
7.17	9.06	7.38	11.66	11.17	14.36	19.38	14.65	12.66	6.04	6.69	8.82
2.02	6.66	5.85	4.34	6.64	9.36	12.85	6.61	9.57	5.76	5.15	7.31
8.78	13.70	11.76	11.57	13.56	10.00	22.64	11.54	6.67	8.47	8.47	11.86
3.47	5.16	4.44	7.18	9.18	9.70	16.07	12.05	12.20	7.72	8.04	9.79
4.76	8.50	7.35	7.70	9.79	9.69	17.19	10.07	9.48	7.32	7.22	9.65
0.344	0.854	0.992	0.157	0.550	0.034	0.584	0.089	0.166	0.274	0.748	0.613

promoter

131	132	133	134	135	136	137	138	139	140	141	142
28154212	28154217	28154226	28154258	28154264	28154267	28154273	28154301	28154303	28154317	28154322	28154336
<1	<1	<1	9.49	<1	<1	<1	<1	<1	<1	<1	<1
<1	<1	<1	<1	<1	<1	1.75	<1	<1	<1	<1	<1
1.21	<1	<1	<1	<1	<1	<1	<1	<1	<1	<1	<1
<1	<1	<1	<1	<1	<1	<1	<1	<1	<1	<1	<1
<1	<1	<1	<1	<1	<1	<1	<1	<1	<1	<1	<1
<1	<1	<1	<1	<1	<1	<1	<1	<1	<1	<1	<1
8.12	9.43	6.55	9.51	16.07	10.26	11.85	5.93	7.78	6.96	4.61	4.47
9.75	9.79	4.75	7.93	12.94	8.70	10.21	5.12	8.33	7.11	3.26	6.17
8.17	9.72	6.91	11.12	18.01	12.17	14.29	5.23	7.77	9.40	4.58	4.42
8.68	9.65	6.07	9.52	15.67	10.38	12.12	5.42	7.96	7.82	4.15	5.02
6.87	7.73	3.29	9.80	11.51	9.30	10.09	5.94	5.94	4.78	2.27	4.47
16.98	16.67	13.92	12.45	12.45	9.83	11.35	6.75	6.75	5.68	2.06	5.21
8.75	8.37	3.49	11.18	15.68	8.83	11.33	6.48	7.86	6.38	3.75	4.60
10.87	10.92	6.90	11.15	13.22	9.32	10.92	6.39	6.85	5.61	2.69	4.76
0.526	0.680	0.827	0.246	0.275	0.369	0.394	0.049	0.130	0.074	0.104	0.696

TALE Target

143	144	145	146	147	148	149	150	151	152	153	154
28154347	28154351	28154413	28154421	28154423	28154426	28154453	28154472	28154476	28154483	28154485	28154490
<1	<1	<1	<1	<1	<1	<1	<1	<1	<1	<1	<1
<1	<1	<1	<1	<1	<1	<1	<1	<1	<1	<1	<1
<1	<1	<1	<1	<1	<1	<1	<1	<1	<1	<1	<1
<1	<1	<1	<1	<1	<1	<1	<1	<1	<1	<1	<1
<1	<1	<1	<1	<1	<1	<1	<1	<1	<1	<1	<1
<1	<1	<1	<1	<1	<1	<1	<1	<1	<1	<1	<1
4.43	3.35	4.69	22.65	19.62	15.20	13.30	11.40	7.42	4.56	5.44	15.15
3.74	4.01	3.90	15.25	13.85	12.17	10.48	11.20	6.70	4.53	3.29	14.45
2.84	4.62	3.62	17.95	14.74	13.82	10.77	10.88	8.10	3.50	3.73	17.03
3.67	3.99	4.07	18.62	16.07	13.73	11.52	11.16	7.41	4.20	4.15	15.54
5.41	5.18	3.02	8.03	8.03	6.61	5.47	7.99	5.74	2.30	4.83	10.47
7.14	5.40	5.73	3.77	3.77	4.69	4.35	9.64	5.29	2.34	3.91	11.57
3.91	3.96	4.47	14.83	11.88	10.57	7.68	9.04	8.70	2.21	4.32	16.49
5.49	4.85	4.41	8.88	7.89	7.29	5.83	8.89	6.58	2.28	4.35	12.85
0.156	0.216	0.712	0.066	0.050	0.029	0.013	0.011	0.509	0.005	0.791	0.250

155	156	157	158	159	160
28154498	28154517	28154519	28154523	28154528	28154536
<1	<1	<1	<1	<1	<1
<1	<1	<1	1.07	<1	<1
<1	<1	<1	<1	<1	<1
<1	<1	<1	<1	<1	<1
<1	<1	<1	<1	<1	<1
<1	<1	<1	<1	<1	<1
3.91	5.53	2.22	4.05	10.97	5.73
3.86	4.49	2.70	5.72	8.63	5.88
4.79	4.95	2.40	4.11	10.31	4.43
4.19	4.99	2.44	4.62	9.97	5.35
3.54	5.35	2.26	4.54	8.80	5.41
4.37	3.28	3.07	7.93	12.68	4.71
4.11	1.70	1.39	3.48	8.92	3.63
4.01	3.44	2.24	5.32	10.13	4.58
0.673	0.232	0.712	0.658	0.918	0.331

Supplementary Table 2. Primers used for bilsuflite sequencing of *Ascl1*

Primer name	Sequence	Tm
RatAscl1_3UTR_forward	TGAAGTGTATTTGTTTTTAAAGTTTATTTTTAGTAGGG	
RatAscl1_3UTR_reverse	AAATCAACRACCAAACAAATCAAAC	56
RatAscl1_Exon2_F	ATATGTTTTYGGAGGGTGGTAAAGTTTAGG	
RatAscl1_Exon2_R	CCRAAACTAATTTCTCCCCTCTTC	58
RatAscl1_exon1_F	TTAGGGGAYGTAGAGAGTTAGAAGGAAG	
RatAscl1_exon1_R	CAACCRCCCAACACTCTCTCACTTCTAAC	60
RatAscl1_promoter_F	TTAYGTTTTTTGGTTAGAAGTGAGAGAGTGTTGGG	
RatAscl1_promoter_R	CTTTCCCTCCAAACTTTCTACCTAAAAAACCCAAATC	60

Supplementary Table 3: PCR primers to incorporate the inserts into the vector. The restriction sites are highlighted in green, HA tag is represented in blue font, and sequences complementary to the inserts are presented in black font.

Insert	Forward	Reverse
**TALE**	acttaaGGATCCgccaccATGTACCCATACGATGTTCCAGATTACGCTTCGCGGACCCGGCTCCCTTCC	cgatgcGCTAGCTGACGCGCGAGTTTGGTCCCCTTCATGCAT
**TET1CD**	cgatgcGGTACCgccaccATGGAACTGCCCACCTGCAGCTGTCTTGATC	acttaaGGATCCGACCCAATGGTTATAGGGCCCC
**DNMT3ACD**	cgatgcGGTACCgccaccATGCCAGCTGAGAAGAGGAAGCCCATCC	acttaaGGATCCCACACAAGCAAAATATTCCTTCAG

Supplementary Table 4: Primers used in this study for sequencing the TALE fusion-proteins

TALE1-CIB1-mCherry fusion protein			
**Primer name**	**Sequence (5' to 3')**	**Primer name**	**Sequence (3' to 5')**
**TALE_SP1**	GAGGGGTTTTATGCGATG	**TALE_SP4**	TGCCACTCGATGTGATGTCCTC

**TALE_SP2**	CCAGTTGCTGAAGATCGCGAAGC	**TALE_SP5**	CCCTCCCGTAATCATAGA
**TALE_SP3**	ACTTACACCCGAACAAGTCG	**TALE_SP6**	AAAGGCATTAAAGCAGCGTATCC
**TET1CD-CRY2PHR-EGFP (TCE) fusion-protein**			
**TCE_SP1**	GAGGGGTTTTATGCGATG	**TCE_SP3**	GAGTGTGCCCGGCGAGAG
**TCE_SP2**	ACCAGCCCTCCTTCCTCAC	**TCE_SP4**	CAGATAAAGACAGGGAATACCG
**DNMT3ACD-CRY2PHR-EGFP (DCE) fusion-protein**			
**DCE_SP1**	GAGGGGTTTTATGCGATG	**DCE_SP3**	CGGTATTCCCTGTCTTTATCTG
**DCE_SP2**	ATGACCTCTCCATTGTCAAC	**DCE_SP4**	GCTCGGCAGTGACGCCCTT

Supplementary Table 5: Primers used in this study for bisulfite conversion PCR (BSP) of *Ascl1*

Region	Forward	Reverse	Product size (bp)	Tm
Ascl1 promoter	**5'-/5BiosG** /GGGAGGTTGTAGTTTTTATTT	ATTTCTAAAACCACCCCCTAAC	216	57
Ascl1 exon	Qiagen Assay # PM00574812		85	50

Supplementary Table 6: Analyzed sequence and the sequencing primers for pyro-sequencing; all primers listed are 5' to 3'

Region	Sequenced CpG region	Sequencing primer (5' to 3')
Ascl1 promoter	RACTAAATAAAAAATTTACA AAAAACCRACRCRAACAA	ACTACAACAACAAACCC
Ascl1 Exon	TTTYGGGAGTATGTTTTTAA YGGYGT	N/A

Y/ R (complement of Y) are the potential methylated cytosines at the target CpG sites.

Supplementary sequence-1: TALE-1 (Ascl1)-CIB1-mCherry (~173.24 kD)

MYPYDVPDYASRTRLPSPPAPSPAFSADSFSDLLRQFDPSLFNTSLFDSLPPFGAHHTEAATGE
WDEVQSGLRAADAPPPTMRVAVTAARPPRAKPAPRRRAAQPSDASPAAQVDLRTLGYSQQQQEK
IKPKVRSTVAQHHEALVGHGFTHAHIVALSQHPAALGTVAVKYQDMIAALPEATHEAIVGVGKQ
WSGARALEALLTVAGELRGPPLQLDTGQLLKIAKRGGVTAVEAVHAWRNALTGAPLNLTPEQVV
AIASHDGGKQALETVQRLLPVLCQAHGLTPEQVVAIASHDGGKQALETVQRLLPVLCQAHGLTP
EQVVAIASHDGGKQALETVQRLLPVLCQAHGLTPEQVVAIASNNGGKQALETVQRLLPVLCQAH
GLTPEQVVAIASNNGGKQALETVQRLLPVLCQAHGLTPEQVVAIASHDGGKQALETVQRLLPVL
CQAHGLTPEQVVAIASNGGGKQALETVQRLLPVLCQAHGLTPEQVVAIASNNGGKQALETVQRL
LPVLCQAHGLTPEQVVAIASNIGGKQALETVQRLLPVLCQAHGLTPEQVVAIASNIGGKQALET
VQRLLPVLCQAHGLTPEQVVAIASNGGGKQALETVQRLLPVLCQAHGLTPEQVVAIASNIGGKQ
ALETVQRLLPVLCQAHGLTPEQVVAIASNIGGKQALETVQRLLPVLCQAHGLTPEQVVAIASNI
GGKQALETVQRLLPVLCQAHGLTPEQVVAIASHDGGKQALETVQRLLPVLCQAHGLTPEQVVAI
ASNIGGKQALETVQRLLPVLCQAHGLTPEQVVAIASNNGGKQALETVQRLLPVLCQAHGLTPEQ
VVAIASNNGGKQALETVQRLLPVLCQAHGLTPEQVVAIASHDGGRPALESIVAQLSRPDPALAA
LTNDHLVALACLGGRPALDAVKKGLPHAPALIKRTNRRIPERTSHRVADHAQVVRVLGFFQCHS
HPAQAFDDAMTQFGMSRHGLLQLFRRVGVTELEARSGTLPPASQRWDRILQASGMKRAKPSPTS
TQTPDQASLHAFADSLERDLDAPSPMHEGDQTRASASGGGGSGGGGSNGAIGGDLLLNFPDMSV
LERQRAHLKYLNPTFDSPLAGFFADSSMITGGEMDSYLSTAGLNLPMMYGETTVEGDSRLSISP
ETTLGTGNFKAAKFDTETKDCNEAAKKMTMNRDDLVEEGEEEKSKITEQNNGSTKSIKKMKHKA
KKEENNFSNDSSKVTKELEKTDYIHVRARRGQATDSHSIAERVRREKISERMKFLQDLVPGCDK
ITGKAGMLDEIINYVQSLQRQIEFLSMKLAIVNPRPDFDMDDIFAKEVASTPMTVVPSPEMVLS
GYSHEMVHSGYSSEMVNSGYLHVNPMQQVNTSSMVSKGEEDNMAIIKEFMRFKVHMEGSVNGHE
FEIEGEGEGRPYEGTQTAKLKVTKGGPLPFAWDILSPQFMYGSKAYVKHPADIPDYLKLSFPEG
FKWERVMNFEDGGVVTVTQDSSLQDGEFIYKVKLRGTNFPSDGPVMQKKTMGWEASSERMYPED
GALKGEIKQRLKLKDGGHYDAEVKTTYKAKKPVQLPGAYNVNIKLDITSHNEDYTIVEQYERAE
GRHSTGGMDELYK*

Supplementary sequence-2: TET1CD-linker-importin αNLS-CRY2PHR-NLS-VP64AD-2A-EGFP (175.18 kD)

MELPTCSCLDRVIQKDKGPYYTHLGAGPSVAAVREIMENRYGQKGNAIRIEIVVYTGKEGKSSH
GCPIAKWVLRRSSDEEKVLCLVRQRTGHHCPTAVMVVLIMVWDGIPLPMADRLYTELTENLKSY
NGHPTDRRCTLNENRTCTCQGIDPETCGASFSFGCSWSMYFNGCKFGRSPSPRRFRIDPSSPLH
EKNLEDNLQSLATRLAPIYKQYAPVAYQNQVEYENVARECRLGSKEGRPFSGVTACLDFCAHPH
RDIHNMNNGSTVVCTLTREDNRSLGVIPQDEQLHVLPLYKLSDTDEFGSKEGMEAKIKSGAIEV
LAPRRKKRTCFTQPVPRSGKKRAAMMTEVLAHKIRAVEKKPIPRIKRKNNSTTTNNSKPSSLPT
LGSNTETVQPEVKSETEPHFILKSSDNTKTYSLMPSAPHPVKEASPGFSWSPKTASATPAPLKN
DATASCGFSERSSTPHCTMPSGRLSGANAAAADGPGISQLGEVAPLPTLSAPVMEPLINSEPST
GVTEPLTPHQPNHQPSFLTSPQDLASSPMEEDEQHSEADEPPSDEPLSDDPLSPAEEKLPHIDE
YWSDSEHIFLDANIGGVAIAPAHGSVLIECARRELHATTPVEHPNRNHPTRLSLVFYQHKNLNK
PQHGFELNKIKFEAKEAKNKKMKASEQKDQAANEGPEQSSEVNELNQIPSHKALTLTHDNVVTV

SPYALTHVAGPYNHWVGSATMKRPAATKKAGQAKKKKMDKKTIVWFRRDLRIEDNPALAAAAHE
GSVFPVFIWCPEEEGQFYPGRASRWWMKQSLAHLSQSLKALGSDLTLIKTHNTISAILDCIRVT
GATKVVFNHLYDPVSLVRDHTVKEKLVERGISVQSYNGDLLYEPWEIYCEKGKPFTSFNSYWKK
CLDMSIESVMLPPPWRLMPITAAAEAIWACSIEELGLENEAEKPSNALLTRAWSPGWSNADKLL
NEFIEKQLIDYAKNSKKVVGNSTSLLSPYLHFGEISVRHVFQCARMKQIIWARDKNSEGEESAD
LFLRGIGLREYSRYICFNFPFTHEQSLLSHLRFFPWDADVDKFKAWRQGRTGYPLVDAGMRELW
ATGWMHNRIRVIVSSFAVKFLLLPWKWGMKYFWDTLLDADLECDILGWQYISGSIPDGHELDRL
DNPALQGAKYDPEGEYIRQWLPELARLPTEWIHHPWDAPLTVLKASGVELGTNYAKPIVDIDTA
RELLAKAISRTREAQIMIGAAPASSPKKKRKVEASGSGRADALDDFDLDMLGSDALDDFDLDML
GSDALDDFDLDMLGSDALDDFDLDMLINSRGSGEGRGSLLTCGDVEENPGPVSKGEELFTGVVP
ILVELDGDVNGHKFSVSGEGEGDATYGKLTLKFICTTGKLPVPWPTLVTTLTYGVQCFSRYPDH
MKQHDFFKSAMPEGYVQERTIFFKDDGNYKTRAEVKFEGDTLVNRIELKGIDFKEDGNILGHKL
EYNYNSHNVYIMADKQKNGIKVNFKIRHNIEDGSVQLADHYQQNTPIGDGPVLLPDNHYLSTQS
ALSKDPNEKRDHMVLLEFVTAAGITLGMDELYK*

Supplementary sequence-3: DNMT3ACD-linker-importin αNLS-CRY2PHR-NLS-VP64AD-2A-EGFP (128.67 kD)

MPAEKRKPIRVLSLFDGIATGLLVLKDLGIQVDRYIASEVCEDSITVGMVRHQGKIMYVGDVRS
VTQKHIQEWGPFDLVIGGSPCNDLSIVNPARKGLYEGTGRLFFEFYRLLHDARPKEGDDRPFFW
LFENVVAMGVSDKRDISRFLESNPVMIDAKEVSAAHRARYFWGNLPGMNRPLASTVNDKLELQE
CLEHGRIAKFSKVRTITTRSNSIKQGKDQHFPVFMNEKEDILWCTEMERVFGFPVHYTDVSNMS
RLARQRLLGRSWSVPVIRHLFAPLKEYFACVGSATMKRPAATKKAGQAKKKKMDKKTIVWFRRD
LRIEDNPALAAAAHEGSVFPVFIWCPEEEGQFYPGRASRWWMKQSLAHLSQSLKALGSDLTLIK
THNTISAILDCIRVTGATKVVFNHLYDPVSLVRDHTVKEKLVERGISVQSYNGDLLYEPWEIYC
EKGKPFTSFNSYWKKCLDMSIESVMLPPPWRLMPITAAAEAIWACSIEELGLENEAEKPSNALL
TRAWSPGWSNADKLLNEFIEKQLIDYAKNSKKVVGNSTSLLSPYLHFGEISVRHVFQCARMKQI
IWARDKNSEGEESADLFLRGIGLREYSRYICFNFPFTHEQSLLSHLRFFPWDADVDKFKAWRQG
RTGYPLVDAGMRELWATGWMHNRIRVIVSSFAVKFLLLPWKWGMKYFWDTLLDADLECDILGWQ
YISGSIPDGHELDRLDNPALQGAKYDPEGEYIRQWLPELARLPTEWIHHPWDAPLTVLKASGVE
LGTNYAKPIVDIDTARELLAKAISRTREAQIMIGAAPASSPKKKRKVEASGSGRADALDDFDLD
MLGSDALDDFDLDMLGSDALDDFDLDMLGSDALDDFDLDMLINSRGSGEGRGSLLTCGDVEENP
GPVSKGEELFTGVVPILVELDGDVNGHKFSVSGEGEGDATYGKLTLKFICTTGKLPVPWPTLVT
TLTYGVQCFSRYPDHMKQHDFFKSAMPEGYVQERTIFFKDDGNYKTRAEVKFEGDTLVNRIELK
GIDFKEDGNILGHKLEYNYNSHNVYIMADKQKNGIKVNFKIRHNIEDGSVQLADHYQQNTPIGD
GPVLLPDNHYLSTQSALSKDPNEKRDHMVLLEFVTAAGITLGMDELYK*
